# Simply effective? The differential effects of solution-focused and problem-focused coaching questions in a self-coaching writing exercise

**DOI:** 10.3389/fpsyg.2022.895439

**Published:** 2022-08-18

**Authors:** Lara Solms, Jessie Koen, Annelies E. M. van Vianen, Tim Theeboom, Bianca Beersma, Anne P. J. de Pagter, Matthijs de Hoog

**Affiliations:** ^1^Department of Work and Organizational Psychology, University of Amsterdam, Amsterdam, Netherlands; ^2^Department of Pediatrics, Erasmus MC-Sophia Children’s Hospital, Erasmus Medical Center Rotterdam, Rotterdam, Netherlands; ^3^Department of Sustainable Productivity and Employability, Netherlands Organization for Applied Scientific Research, Leiden, Netherlands; ^4^School of Business and Economics, Vrije Universiteit Amsterdam, Amsterdam, Netherlands; ^5^Department of Organization Sciences, Vrije Universiteit Amsterdam, Amsterdam, Netherlands; ^6^Department of Pediatrics, Willem-Alexander Children’s Hospital, Leiden University Medical Center, Leiden, Netherlands

**Keywords:** coaching questions, self-coaching, solution-focused coaching, problem-focused coaching, affect, self-efficacy, goal orientation, action planning

## Abstract

Coaching is a systematic and goal-oriented one-on-one intervention by a coach aimed to guide clients in their professional and personal development. Previous research on coaching has demonstrated effects on a number of positive outcomes, including well-being and performance, yet little is known about the processes that underlie these outcomes, such as the type of questions coaches use. Here, we focus on three different types of coaching questions, and aim to uncover their immediate and sustained effects for affect, self-efficacy, and goal-directed outcomes, using a between-subjects experiment. One hundred and eighty-three medical residents and PhD students from various medical centers and healthcare organizations in the Netherlands were recruited to participate in a self-coaching writing exercise, where they followed written instructions rather than interacting with a real coach. All participants were randomly allocated to one of three conditions: either one of two solution-focused coaching conditions (i.e., the success or miracle condition) or a problem-focused coaching condition. Self-report questionnaires were used to measure key outcomes of coaching, that is positive and negative affect, self-efficacy, goal orientation, action planning (i.e., quantity and quality) and goal attainment. Two follow-up measurements assessed if the effects of the self-coaching exercise led to problem-solving actions within an initial follow-up period of 14 days and a subsequent follow-up period of 10 days. Findings showed that participants experienced more positive affect, less negative affect, and higher approach goal orientation after the solution-focused coaching exercise compared to the problem-focused coaching exercise. In all conditions, goal attainment increased as a consequence of the self-coaching intervention. We discuss the implications of our findings for the science and practice of contemporary coaching.

## Introduction

*Problem talk creates problems, solution talk creates solutions – Steve de Shazer (Berg and Szabo, 2005).*

In the past two decades, the field of psychology has largely shifted its focus from (curing) mental illness to (promoting) well-being. With that shift, a new field of research and practice has emerged, that of positive psychology. The field of positive psychology is, in essence, the study of positive human functioning or happiness as defined by the presence of positive emotions, engagement and meaning (Seligman and Csikszentmihalyi, 2000). Instead of fixing what is broken, applied positive psychology highlights what is working well in people’s lives, and uncovers and amplifies people’s individual strengths, hopes, and positive virtues. The theory of positive psychology, through its strength-based approach to human functioning, is the basis of coaching research and practice (Kauffman, 2006).

With a growing attention to individual well-being and thriving, professional coaching has become a popular intervention at both the workplace and the private domain. Coaching can be defined as “a result-oriented, systematic process in which the coach facilitates the enhancement of life experience and goal attainment in the personal and/or professional lives of normal, non-clinical clients” (Grant, 2003, p. 254). Given the continuous need of employees to adapt to the changing nature of work and organizations –which can be demanding and a risk factor for well-being and health (George and Jones, 2001; van den Heuvel et al., 2013; Johnston, 2018)– employees increasingly seek the support of a coach to help them deal with the many challenges that working life can present. With this uptake, coaching as a profession also continues to keep growing: The International Coach Federation, the most recognized governing body for coaches around the world, counts more than 40,000 members in 151 countries in 2021 (International Coach Federation, 2021), but this is a very conservative estimate of the number of people actually working as coaches. Research on the effects of coaching supports its popularity: studies have repeatedly demonstrated the positive effects of coaching on both well-being (e.g., reducing stress and burnout) and performance outcomes (e.g., goal attainment; Theeboom et al., 2014; Jones et al., 2016; McGonagle et al., 2020; Solms et al., 2021).

Although research on coaching has accelerated in the past years, there is still a lot that we do not know. First, relatively little is known about the coaching techniques and psychological mechanisms underlying positive coaching outcomes. Here, we aim to uncover these mechanisms by focusing on the effects of three different questioning techniques that coaches can use (Bozer and Jones, 2018; Fontes and Dello Russo, 2021; Jones et al., 2021). We employ a self-coaching intervention rather than a real-life coaching intervention to examine the effects of each questioning technique and isolate it from relational factors that might otherwise impact the outcomes of coaching. That is, in real-life coaching, coaches tend to use a blend of different questioning techniques, and relational factors such as similarity attraction (i.e., similarity between coach and client may increase liking for one another) may play a role in their effectiveness. Employing an experimental design with a self-coaching exercise allows us to eliminate such confounding, relational factors and uncover the unique effects of each coaching question on coaching outcomes and their underlying psychological mechanisms. Nevertheless, we note that self-coaching is different from real-life coaching where a professional coach guides the coachee in a systematic, and goal-oriented fashion to goal-attainment and personal change. Second, the majority of experimental studies have focused on the immediate effects of coaching questions (Theeboom et al., 2014). Given that coaching is a temporary investment, it is important to investigate if coaching questions can foster goal-directed change (e.g., action planning) beyond such immediate effects. Here, we examine both the immediate effects of questioning techniques and their effects during a brief follow-up period. Third and finally, existing experimental research on the effectiveness of coaching –the method that supports drawing causal conclusions– has exclusively been conducted among undergraduates (e.g., Grant, 2012; Theeboom et al., 2016; Grant and O’Connor, 2018). This is unfortunate, given that the majority of coaching takes place within an organizational context. It is thus crucial to conduct experimental research among working individuals and in a context in which coaching normally takes place. Because we use an experimental design in which we test different self-coaching techniques among medical professionals, this study combines the advantage of experimental control with higher ecological validity, allowing stronger generalization of findings to real-life coaching of medical professionals.

### Problem-focused and solution-focused coaching

Questions are an integral part of any coaching conversation (Grant and O’Connor, 2010). Here, we distinguish between questioning techniques that have their roots in *problem-focused coaching* and questioning techniques that have their roots in *solution-focused coaching*. Problem-focused coaching approaches originate from more traditional, generally psychotherapy-inspired schools that tend to focus their questioning on the client’s problem. Although positive psychology provides a solid theoretical and practical backbone to the science and practice of coaching (Kauffman, 2006; Seligman, 2007), numerous coaching practitioners are rooted in the therapeutic model which concentrates on repairing damage rather than boosting strengths (Kauffman and Scoular, 2004; Kauffman, 2006). Consequently, these coaches tend to use questioning techniques that are aimed to understand (and eliminate) the client’s problem (i.e., problem-focused questioning techniques^1^). These questions can for instance be focused on the origin of a problem: “How long has this been a problem? How did it start?” (Grant, 2003, p. 26). By analyzing the root cause of a problem and how it manifests in dysfunctional patterns and behavior, coach and client work toward a global understanding of the origin of the problem and its consequences (Lee, 2010). In contrast, coaches with a deep rootedness in strength-based approaches tend to focus on nurturing clients’ positive skills and qualities. Consequently, and in line with the premises of positive psychology they tend to use questioning techniques that activate existing resources and prioritize solution building over problem solving (i.e., solution-focused questioning techniques; O’Connell et al., 2013). These questions can for instance be focused on exploring previous solutions (“Can you think of a time when you managed a similar problem well? What did you do?”) or exceptions to the problem (“Has there been a time where this problem was not present?”).

Stemming from *Solution-Focused Brief Therapy* (De Shazer, 1988), solution-focused coaching represents a paradigm shift focusing on what is already working well in a client’s life (O’Connell et al., 2013) rather than focusing predominantly on the problem and its origin. In practice, the problem that has brought the client to coaching in the first place will almost always be the starting point of any coach conversation and as such, problem-focused approaches play an important role especially at the beginning of the coaching process. While problem-focused coaching addresses solutions relatively late in the process, in Solution-Focused Brief Therapy and coaching, solutions are developed relatively quickly by focusing on strategies and behavior that has been proven helpful instead of focusing on a client’s dysfunctional behavior (De Shazer, 1988). By identifying occasions in a client’s life where the problem could have occurred but did not (referred to as “exception times”), coach and client can work toward solutions without spending too much time on the problem itself. Research in various populations (e.g., university students, patients, managers) has shown that solution-focused approaches correlate with well-being and promote goal pursuit (Green et al., 2006; Grant, 2014; Pakrosnis and Cepukiene, 2015; Zhang et al., 2018), a finding that has also been confirmed in a meta-analysis on coaching in organizational and educational settings (Theeboom et al., 2014).

While problem-focused coaching centers around asking questions about the client’s problem, solution-focused coaching can use different types of questions: the miracle question or success question are prototypical examples. The miracle question lets clients imagine a situation in which the problem miraculously no longer exists (De Shazer and Dolan, 2012). This questioning technique uses mental imagery to stir the conversation away from the problem toward a desired situation where the problem is absent. Applying this technique can be an eyeopener for clients who tend to focus primarily on the struggles they encounter, and consequently pave the way for change (De Shazer and Dolan, 2012). The success question lets clients think back to previous situations in which they have successfully managed a problem. This questioning technique is based on the assumption that people have solved plenty of problems in the course of their life and are therefore able to generate successful strategies to solve their current problems (De Shazer and Dolan, 2012). This idea strongly resembles Bandura’s (1991) concept of self-efficacy: a person’s belief in his or her capability to successfully perform a particular task. Such self-efficacy beliefs are strongly influenced by past experiences of success (i.e., mastery experiences). As such, the success question can make past mastery experiences salient to the client and increase their sense of competence.

Despite its strong roots in seminal theory (e.g., social cognitive theory; Bandura, 1991) and its frequent use in practice, the success question has not received much scientific attention. This is unfortunate, because a deeper understanding of the mechanisms through which specific questioning techniques can improve client outcomes would not only advance theory in the field of coaching but would also allow practitioners to resort to coaching techniques that are tailored to and more effective for their clients (Grant, 2020). In this study, we will therefore examine the effects of the success question in addition to the miracle question and will compare these effects with those of the problem-focused question.

### Theoretical background and hypotheses

In line with positive psychology theory and common definitions of coaching as a change process aimed at building personal strengths and attaining personal goals, here we focus on key variables relevant in the context of goal-directed self-regulation: affect, self-efficacy, goal-orientation, goal pursuit, and problem-solving actions.

#### Question focus and affect

Research comparing problem-focused with solution-focused questioning paints a more positive picture in favor of the solution-focused approach (e.g., Braunstein and Grant, 2016; Theeboom et al., 2016). Specifically, solution-focused questions (as compared to problem-focused questions) may increase positive affective states (e.g., feeling energetic) and may decrease negative affective states (e.g., feeling anxious; Theeboom et al., 2016; Grant and O’Connor, 2018). According to positive psychology theory (Seligman et al., 2005), when people are encouraged to think about a desired outcome in the future or past successes –rather than directing their attention to the problem– they will likely experience positive emotions (such as feeling energetic or calm) that accompany these thoughts. This idea is supported by regulatory focus theory (Higgins, 2002), proposing that goals aimed at achieving positive outcomes (rather than at avoiding negative outcomes) are linked to positive emotions (Idson et al., 2000). In contrast, goals aimed at avoiding or overcoming negative outcomes are linked to negative emotions. Hence, when people focus on potential solutions, they will experience positive emotions, whereas when they mainly focus on their problem, they will feel increased discomfort and negative emotions (Theeboom et al., 2016). Based on this theorizing, we generate our first hypothesis.

**Hypothesis 1:** Compared to problem-focused questioning, solution-focused questioning leads to (a) higher positive affect and (b) lower negative affect.

#### Question focus and self-efficacy

Coaches often seek to increase their clients’ self-efficacy to promote a sense of personal agency and goal attainment (Grant, 2012). This idea is rooted in Bandura’s (1977) social learning theory that posits that past experiences guide people’s future actions and that people engage in actions that have proven useful in the past. In solution-focused coaching, self-efficacy is promoted by focusing on “what is going well” instead of “what is going wrong.” Under the tenet “If it works, do more of it” therapists and coaches encourage clients to engage in activities that have been proven useful. Small steps in the right direction will likely spark further steps, gradually leading the client to feel “better enough” to end therapy or coaching (De Shazer and Dolan, 2012, p. 2).

The miracle question is typically used by coaches to spark optimism of a hopeful future and break free from existing –often dysfunctional– cognitive patterns and beliefs (Grant and O’Connor, 2010, 2018; Braunstein and Grant, 2016). By encouraging the client to envision a world without the problem, people are reminded of their qualities and skills that have been overshadowed by the seeming incompetence to handle the problem successfully. Therefore, the miracle question (as opposed to a problem-focused coaching question) likely increases self-efficacy to solve a personal problem. Moreover, we expect that the solution-focused success question will result in even higher self-efficacy than the solution-focused miracle question, because the success question instructs clients to think about previous mastery experiences, which –according to Bandura’s social learning theory– should be particularly strongly related to self-efficacy (Bandura, 1982).

**Hypothesis 2a:** Compared to problem-focused questioning, solution-focused questioning leads to higher self-efficacy.

**Hypothesis 2b:** Compared to the solution-focused miracle question, the solution-focused success question leads to higher self-efficacy.

#### Question focus and goal orientation

With goal pursuit lying at the heart of coaching interventions, coaches may seek to assist clients in formulating effective goals, that is, approach rather than avoidance goals (Elliot and Church, 1997; Elliot et al., 1997). Solution-focused questioning can help to achieve this as it emphasizes a desired outcome that one aims to achieve (i.e., an approach goal) rather than a negative outcome that one aims to avoid (i.e., an avoidance goal). This is in line with the self-regulation model by Carver and Scheier (1998) proposing that behavioral regulation with negative reference points (i.e., an undesired end state) is less fruitful than behavioral regulation with positive reference points (i.e., a desired end state) because the former fails to provide clients with a clear direction. Instead of focusing on the things that are going wrong, solution-focused coaching rather emphasizes behaviors that proved beneficial for the client during times of improvement (De Shazer and Dolan, 2012). Drawing on the hierarchical model of approach-avoidance motivation (Elliot, 2006) we argue that solution-focused coaching –due to its strong focus on positive outcomes and how to attain them– is inherently associated with an approach rather than avoidance orientation. Specifically, both the miracle and the success question draw attention to a desired outcome that either has “magically” come about (i.e., the miracle question) or has previously been achieved (i.e., the success question). As such, we hypothesize that the solution-focused coaching questions will stimulate approach goal orientation and inhibit avoidance goal orientation.

**Hypothesis 3:** Compared to problem-focused coaching, solution-focused coaching leads to (a) higher approach goal orientation and (b) lower avoidance goal orientation.

#### Question focus and goal pursuit

Compared to problem-focused coaching, solution-focused coaching approaches are stronger future-focused and goal-directed (De Shazer and Dolan, 2012): considerable time is spent on constructing solutions, presumably more than on analyzing the problem that brought a client to coaching in the first place. Consequently, clients can make goal progress relatively quickly (Iveson, 2002). Drawing on hope theory –that emphasizes agency and pathway thinking as central to the process of goal attainment– (Snyder, 2002), we argue that solution-focused coaching activates clients’ sense of agency (i.e., the belief in one’s capacity to initiate and sustain actions or “willpower”) and goal-directed or “pathway” thinking, which likely promotes goal-directed behavior (e.g., development of action plans) and goal attainment. Solution-focused as opposed to problem-focused coaching is expected to be superior in promoting goal progress (e.g., Grant and O’Connor, 2018; Grant and Gerrard, 2020). Based on this theorizing, research indeed found that participants who engaged in a solution-focused coaching exercise listed more action steps to solve a problem than participants in a problem-focused coaching exercise (Grant, 2012). In line with hope theory and earlier empirical findings, we formulate the following hypothesis:

**Hypothesis 4:** Compared to problem-focused questioning, solution-focused questioning will lead to (a) stronger increases in goal attainment and (b) more and higher quality action planning (i.e., number and quality of action steps) directly after the experimental coaching intervention.

#### Question focus and problem-solving actions

Although coaches can facilitate clients’ goal pursuit through formulation of action plans, clients still need to translate their goals and plans into actual behavior (Theeboom et al., 2016). According to the theory of planned behavior (Ajzen, 1991) behavioral intentions (action plans to solve the problem) will promote actual problem-solving behaviors. We therefore also investigate the effects of problem-focused and solution-focused questioning on *reported* problem-solving actions and *actual* problem-solving actions within a brief follow-up period. Specifically, we include an unobtrusive behavioral measure that captures whether participants actually take action to try and solve their problem. Given the previously described benefits of solution-focused questioning on affective (e.g., positive affect), cognitive (e.g., self-efficacy), and behavioral (i.e., action planning and goal attainment) outcomes, we expect that solution-focused (as opposed to problem-focused) questioning will have stronger effects on goal attainment and *reported* problem-solving actions within a follow-up period of 14 days, as well as on *actual* problem-solving actions within a subsequent follow-up period of 10 days.

**Hypothesis 5:** Compared to problem-focused questioning, solution-focused questioning leads to (a) higher *reported* problem-solving actions (i.e., extent of performing action steps), (b) higher goal attainment, and (c) higher *actual* problem-solving actions during follow-up.

## Materials and methods

### Participants and design

Our sample comprised medical residents and MD/PhD students recruited from several medical centers and healthcare institutions throughout the Netherlands. In total, five medical centers as well as two umbrella training and education alliances that include more than 20 medical centers and several healthcare institutions were approached by the authors and shared the study invitation within their network of residents and MD/PhD students. Participants were invited by email to participate in a study on online coaching. Initially, a total number of 232 participants completed the self-coaching exercise that consisted of written instructions concerning a work-related problem.

**FIGURE 1:**
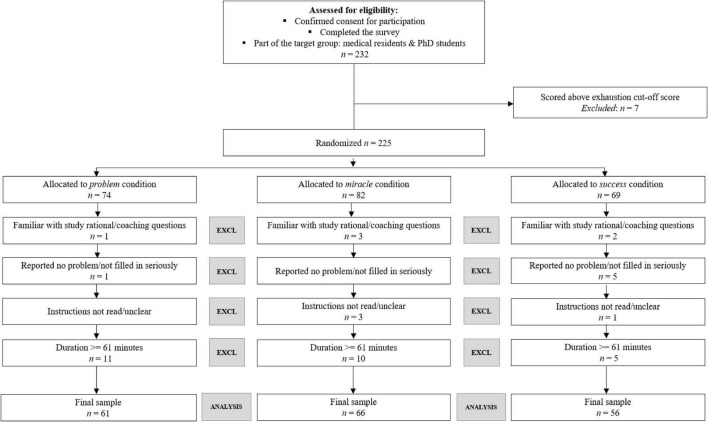
CONSORT flowchart depicting screening and exclusion procedure at T1. EXCL refers to the exclusion of participants. Participants that spend 61 or more minutes on completing the experiment (i.e., extremes based on stem-and-leaf plot) were excluded, because the experimental design requires participants to complete the exercise at once.

In order to preclude any adverse effects of our manipulation on participants’ well-being and in line with coaching operationalized as an intervention for a healthy, non-clinical population, participants were screened at the start of the study on the emotional exhaustion component of the UBOS scale (UBOS; Schaufeli et al., 1996; Theeboom et al., 2016). Because we predicted more positive effects in the solution-focused than in the problem-focused condition, participants who reached a cut-off point of severe exhaustion (cut-off = 4.62; Schaufeli and Van Dierendonck, 2000) were automatically led into one of the two solution-focused coaching conditions. Additionally, these participants were notified at the end of the questionnaire that they scored above average on the exhaustion scale and were advised to seek support from their occupational physician or manager. We excluded their data (*n* = 7) from our analyses. After applying a predetermined exclusion procedure (see Figure 1 for a CONSORT flowchart), our final sample comprised 183 medical residents and medical PhD students (159 residents, 145 females of which 61, 66, and 56 were assigned to the problem, miracle, and success condition, respectively). Their average age was 30.71 (SD = 3.30), ranging from 25 to 46 years.

The study consisted of an online self-coaching writing exercise and questionnaire (T1), a follow-up questionnaire (T2) and an unobtrusive behavioral measure (T3). The self-coaching writing exercise allowed us to test the effects of solution- and problem-focused coaching questions that were experimentally manipulated. Participants were randomly allocated to one of three conditions (problem-focused, solution-focused miracle, or solution-focused success). Including two different types of solution-focused coaching questions (i.e., miracle and success question) allowed us to compare their effects as well rather than merely contrasting solution-focused coaching with problem-focused coaching questions. While the follow-up questionnaire (T2) was used to measure the effects of the coaching exercise (i.e., reported problem-solving actions and goal-attainment) during a brief follow-up period of 14 days, the hidden behavioral measure (T3) aimed to assess actual problem-solving actions.

### Procedure and manipulations

**FIGURE 2:**
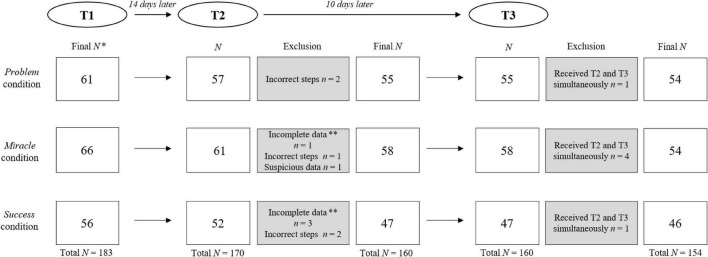
CONSORT flowchart depicting screening and exclusion procedure for T2 and T3 Follow-up. The sample at T1 consisted of 183 participants allocated to one of the three experimental conditions. Of the 183 participants, 1 participant did not indicate their email address and thus did not receive the T2 survey. Of the remaining 182 participants, 170 participants (response rate: 92.9%) filled in the T2 survey. Overall, 10 participants were excluded as they did not complete the survey, indicated that the steps reported were not correct or showed suspicious data entry. The final sample at T2 consisted of 160 participants. The final T3 sample that was analyzed consisted of 154 participants. 6 participants were excluded as they received the link for the website (T3) and the T2 survey simultaneously and this could potentially distort the answers on the T2 survey. *For exclusion procedure at T1, see Figure 1. **Participants that didn’t answer items on effort to perform action steps (but on extent) were included in the sample although these answers were missing.

The study protocol was approved by the Ethics Review Board of the University of Amsterdam. Before starting the online coaching exercise (at T1), participants were informed about the study’s goal and procedure. They were also informed that all data would be handled confidentially, would not be shared with the organizations in which participants were employed, and that participation was completely voluntary. Finally, they read that the study consisted of a self-coaching exercise (T1) and a follow-up questionnaire (T2) they would receive 14 days later. See Figure 2 for details on the exclusion procedure at T2 and T3.

#### Manipulation: Self-coaching writing exercise (T1)

Participants completed the informed consent form, filled in a self-generated identification code to allow matching the T1 and T2 data, provided demographical information (i.e., gender, age, nationality, job position [i.e., medical resident, medical PhD student] and medical specialty, previous experience with coaching and email address for follow-up contact and compensation in the form of an online voucher), and completed the exhaustion screening measure. Participants then started the self-coaching writing exercise. The exercises with the solution-focused miracle or problem-focused questions were based on previous research (Braunstein and Grant, 2016; Theeboom et al., 2016). The exercise with the solution-focused success question was added by the researchers and is based on Bandura’s concepts of self-efficacy and mastery (Bandura, 1982; see the Supplementary material for a detailed description of the self-coaching exercises). As a first step, participants were asked to identify and describe a personal work-related problem that they would like to address during coaching. In order to guarantee a certain degree of standardization of the problems described, we asked participants to describe a problem that related to their job, career or work-life balance that they would like to address in a coaching session. Furthermore, they were asked to report the extent to which the problem was causing discomfort (on a 10-point scale, from 1 [*no discomfort at all*] to 10 [*heavy discomfort*]), and how the problem influenced thoughts and feelings or interfered otherwise with their functioning at work or in their private life. Finally, they were asked to indicate on a scale from 1 (*solution not reached at all*) to 10 (*solution reached*) to what extent they currently had reached the solution to their problem. Hereafter, the manipulation started.

In the problem condition, participants were asked to think back to a day where their problem had been strongly present. Hereafter, they were asked to describe the first thing they had noticed on that day, how they had behaved, thought, and felt in that situation, and how other people had noticed that their problem was strongly present.

In the miracle condition, participants were asked to imagine a situation in which their problem had magically disappeared overnight. They were then asked to describe what they would notice the next morning, how they would behave, think and feel in that situation, and how other people would notice that the problem had disappeared.

In the success condition, participants were asked to think of a situation in the past in which they had experienced the same problem but had been able to manage it successfully. They were asked to describe the first thing they had noticed that day, how they behaved, thought and felt in that situation, and how other people had noticed that they had successfully dealt with the problem.

**FIGURE 3:**
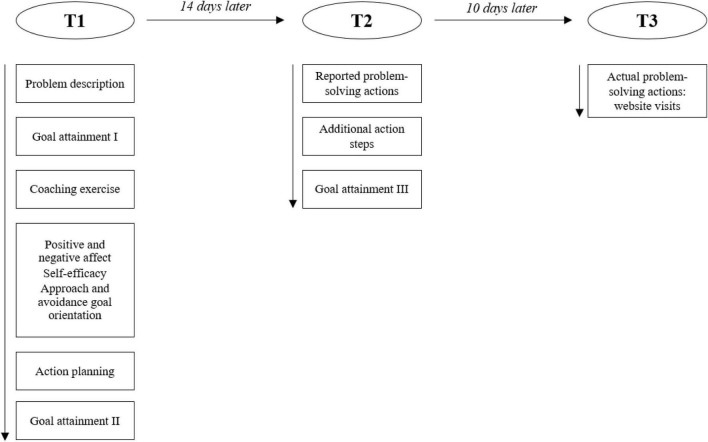
Summary of study design.

Figure 3 presents the experimental procedure as well as the corresponding measures. See the Supplemental Material for information on additional measures.

At T1, participants filled out questionnaires to assess their positive and negative affect, self-efficacy, and goal orientation. Next, their own responses to the self-coaching exercise (i.e., what they had noticed, how they had felt, thought, and behaved) were presented to them and they were asked to list future action steps that would bring them closer to solving their problem. Hereafter, they were asked again about their goal attainment (i.e., how close they felt to the solution of their problem). Finally, participants answered the manipulation check questions, and were thanked for their participation.

At T2, 14 days after completion of the coaching exercise, participants who had completed T1 and had provided their email address received the invitation to the follow-up survey by e-mail. Participants reported their problem-solving actions (i.e., the extent to which participants had performed their action steps described at T1) and goal attainment. At T3, after completion of the T2 measures, they received an invitation to a website providing information on dealing with work-related stress, such as time management and mindfulness. Using a click-through measure, we assessed the number of visits during the upcoming 10 days as an unobtrusive behavioral indicator of participants’ actual (objective) problem-solving actions.

### Measures

Our measures were derived from validated scales and have been used in previous studies in the context of coaching, and beyond. Below, we report reliability indices of our measures, Cronbach’s alpha and McDonalds omega (McNeish, 2018).

#### Emotional exhaustion (T1)

Participants’ emotional exhaustion was measured with the emotional exhaustion subscale of the Dutch version of the Maslach Burnout Inventory adapted for human services such as healthcare (UBOS; Schaufeli et al., 1996; Schaufeli and Van Dierendonck, 2000). The eight items were answered on a seven-point scale ranging from 1 (*never*) to 7 (*always*). An example item is: “Working with people all day is a heavy burden for me” (α = 0.86; ω = 0.86).

#### Goal attainment (T1)

Goal attainment, the extent to which participants had reached the solution to their problem, was measured with a 10-point scale ranging from 1 (*solution not obtained at all*) to 10 (*solution obtained*; see Grant, 2012; Theeboom et al., 2016). Goal attainment was measured before and after the experimental manipulation. The following item was used: “On a scale from 1 to 10, to what extent have you at this point achieved the solution to this problem?”

#### Positive and negative affect (T1)

Participants’ positive and negative affect were measured with the hedonic tone (e.g., “satisfied”; α = 0.89; ω = 0.90), energetic arousal (e.g., “active”; α = 0.82; ω = 0.79), and the tense arousal (e.g., “nervous”; α = 0.90; ω = 0.90) subscales (8 items each) of the UWIST Mood Adjective Checklist (UMACL; Matthews et al., 1990). Participants were asked to indicate on a seven-point scale ranging from 1 (*not applicable at all*) to 7 (*fully applicable*) to what extent these adjectives currently applied to them when thinking about the situation they had just described (i.e., the coaching manipulation).

#### Self-efficacy (T1)

Self-efficacy was measured with the following four items that are based on the Core Self-Evaluations Scale and were adapted to fit the context of the study (CSES; Judge et al., 2004): (1) “I am confident that I can solve my problem”; (2) “If I try my best, I will be able to solve my problem”; (3) “I am full of doubts about my abilities to master my problem”; (4) “I am able to handle my problem well” (α = 0.70; ω = 0.71). The items were answered on a five-point scale ranging from 1 (*completely disagree*) to 5 (*completely agree*).

#### Approach and avoidance goal orientation (T1)

Approach and avoidance goal orientation were measured with three items each, that were based on the Achievement Goal Questionnaire (AGQ; Elliot and Murayama, 2008). We adapted the items to fit the context of the self-coaching exercise. The items were answered on a seven-point scale ranging from 1 (*completely disagree*) to 7 (*completely agree*). Example items of approach and avoidance goal orientation, respectively, are: “I strive to solve my problem as soon as possible” (α = 0.74; ω = 0.74) and “I am going to focus on preventing the problem from getting worse” (α = 0.61; ω = 0.62).

#### Action planning (T1)

Action planning was assessed by asking participants to describe the first steps they would take in the near future to achieve the situation they wished for (i.e., solution of the problem; Grant, 2012). The following item was used: “Can you describe what first small steps you will take in the near future to achieve the desired situation (solution of the problem)?” Fifteen text fields were provided for potential responses. We recorded the number and quality of action steps of each participant by means of four indicators: *specificity*, *uniqueness*, *behavior (i.e., action steps reflect behavior rather than cognitions)*, and *approach goal orientation*. In pairs of two, the authors conducted the coding of the quality indicators based on a coding scheme. See the Supplementary material for a detailed description of the quality criteria and the coding process and scheme.

#### Manipulation check (T1)

With six items that described the nature of the coaching instructions people had received, we assessed whether the manipulation had been successful. Participants rated on a seven-point scale ranging from 1 (*not applicable at all*) to 7 (*fully applicable*) whether the statements were applicable to them. Example items of the problem, miracle, and success conditions, respectively, are: “In this study, I was asked to think about a situation where my problem was very present” (α = 0.71), “In this study I was asked to imagine a situation in which my problem suddenly disappeared.” (α = 0.95), and “In this study, I had to think about what I had done in the past to solve the problem” (α = 0.76).

#### Problem-solving actions (T2)

Participants were shown the personal problem and the action steps they had described during the coaching exercise (at T1). They were asked to indicate to what extent they had performed these steps (on a scale ranging from 1 [*not at all*] to 7 [*completely*]) and how much effort they had spent to do so (on a scale from 1 [*not much*] to 7 [*much*]). Participants then could list additional action steps that had not been listed before. We used the following item: “For each step, indicate to what extent you have performed this step and how much effort you have put into taking this step.”

#### Goal attainment (T2)

Participants indicated on a 10-point scale ranging from 1 (*solution not obtained at all*) to 10 (*solution obtained*) to what extent they had currently reached the solution to their previously described problem. We used the same measure as at T1.

#### Actual problem-solving actions (T3)

Participants received an email with the link to a website providing information that could be useful for dealing with work-related problems (e.g., time management and mindfulness). As an indicator of objective (as opposed to self-reported) problem-solving actions, we used a click-through measure to assess if participants visited the website during a period of 10 days. The specific content displayed on the website can be requested from the first author.

## Results

### Analytical approach

Data were analyzed in SPSS (version 25) using analysis of variance (ANOVA) with condition as between-subjects factor. Significant main effects were followed up with planned contrasts between the problem-focused (coded as −2) and the two solution-focused conditions (coded as 1 each), and –for H2b– between the solution-focused miracle (coded as 1) and the solution-focused success condition (coded as −1). Table 1 shows the means and standard deviations of the key variables in all three conditions. Table 2 presents the correlations of the variables at T1. Table 3 displays a summary of the hypotheses and their results.

**TABLE 1:** Means and standard deviations of the key study variables in all three conditions.

Study variables	Problem condition (*n* = 61)	Miracle condition (*n* = 66)	Success condition (*n* = 56)
	
	Mean (SD)	Mean (SD)	Mean (SD)
**T1: Coaching exercise**			
**Manipulation check**			
Problem items	6.07 (0.85)	3.30 (1.70)	4.54 (1.63)
Miracle items	1.44 (0.73)	6.37 (1.15)	1.71 (1.09)
Success items	3.64 (1.43)	3.00 (1.58)	5.81 (1.18)
PA: hedonic tone	3.85 (1.04)	4.64 (1.22)	4.84 (1.29)
PA: energetic arousal	4.24 (0.92)	4.81 (1.09)	4.83 (0.98)
NA: tense arousal	4.06 (1.15)	3.54 (1.32)	3.52 (1.19)
Self-efficacy	3.48 (0.70)	3.45 (0.73)	3.62 (0.66)
Approach goal orientation	4.77 (1.22)	5.13 (1.02)	5.31 (0.98)
Avoidance goal orientation	5.25 (1.24)	5.38 (1.12)	5.50 (1.02)
Goal attainment pre	4.59 (1.81)	4.77 (1.59)	4.89 (1.89)
Goal attainment post	5.74 (1.77)	5.59 (2.00)	6.14 (2.04)
Number action steps:	3.80 (1.76)	3.89 (1.61)	3.79 (1.59)
**Action steps: quality criteria^a^**			
Specificity	1.62 (0.61)	1.72 (0.66)	1.45 (0.66)
Uniqueness	0.73 (0.24)	0.72 (0.25)	0.68 (0.23)
Behavior	0.77 (0.30)	0.75 (0.30)	0.80 (0.21)
Approach goal orientation	0.90 (0.19)	0.93 (0.14)	0.95 (0.13)
**T2: Follow-up questionnaire^b^**			
Goal attainment	5.71 (1.92)	5.55 (1.74)	5.85 (1.61)
Extent action initiation	3.75 (1.36)	3.72 (1.35)	3.73 (1.34)
Effort action initiation	3.66 (1.74)	3.47 (1.26)	3.11 (1.20)
**T3: Behavioral measure^c^**			
Website visit in %^d^	48.10	57.40	58.70

PA, positive affect, NA, negative affect; Goal attainment pre, before the experimental instructions; Goal attainment post, after the experimental instructions.

^a^Based on n = 58, n = 66, n = 55 for problem condition, miracle condition, and success condition, respectively.

^b^Based on n = 55, n = 58, n = 47 for problem condition, miracle condition, and success condition, respectively, for the goal attainment measure; n = 53, n = 58, n = 46 for problem condition, miracle condition, and success condition, respectively, for the extent measure; n = 45, n = 48, n = 38 for problem condition, miracle condition, and success condition, respectively, for the effort measure.

^c^Based on n = 54, n = 54, n = 46 for problem condition, miracle condition, and success condition, respectively.

^d^Reflects the percentage of participants visiting the website once or more.

**TABLE 2:** Means, standard variations, intercorrelations, and reliabilities of the study variables across the three conditions at T1.

	*M*	SD	1.	2.	3.	4.	5.	6.	7.	8.	9.	10.	11.	12.	13.
1. PA: hedonic tone	4.44	1.25	*(0.89)*												
2. PA: energetic arousal	4.63	1.03	0.71**	*(0.82)*											
3. NA: tense arousal	3.71	1.24	−0.68**	−0.63**	*(0.90)*										
4. Self-efficacy	3.51	0.70	0.30**	0.29**	−0.27**	*(0.70)*									
5. Approach goal orientation	5.07	1.10	0.18*	0.16*	−0.15*	0.35**	*(0.74)*								
6. Avoidance goal orientation	5.37	1.13	–0.00	–0.00	0.09	0.10	0.38**	*(0.61)*							
7. Goal attainment pre	4.75	1.75	0.23**	0.23**	−0.23**	0.46**	0.11	0.03	*(−)*						
8. Goal attainment post	5.81	1.94	0.19*	0.12	−0.17*	0.54**	0.16*	0.02	0.68**	*(−)*					
9. Number action steps	3.83	1.65	–0.02	–0.02	0.16*	–0.04	–0.08	–0.06	0.03	0.08	*(−)*				
Action steps: quality criteria															
10. Specificity	1.61	0.65	0.02	0.03	0.03	0.09	–0.03	–0.01	–0.02	0.07	0.13	*(−)*			
11. Uniqueness	0.71	0.24	0.06	0.03	–0.11	0.06	0.14	–0.11	0.07	0.03	−0.26**	–0.02	*(−)*		
12. Behavior	0.77	0.28	–0.08	–0.14	0.09	–0.02	–0.01	–0.06	0.02	–0.02	0.03	0.16*	–0.07	*(−)*	
13. Approach goal orientation	0.93	0.16	–0.00	–0.04	0.05	0.05	0.06	–0.06	–0.06	0.03	0.10	0.14	–0.08	0.28**	*(−)*

N = 183 for variables 1–9. N = 179 for variables 10–13. Cronbach’s alpha reliability indices are displayed on the diagonal between brackets.*p < 0.05. **p < 0.01.

**TABLE 3:** Summary of hypotheses and results.

Hypothesis	Description	Result
H1a	Compared to problem-focused questioning, solution-focused questioning leads to higher positive affect	*Supported*
H1b	Compared to problem-focused questioning, solution-focused questioning leads to lower negative affect	*Supported*
H2a	Compared to problem-focused questioning, solution-focused questioning leads to higher self-efficacy	*Not supported*
H2b	Compared to the solution-focused miracle question, the solution-focused success question leads to higher self-efficacy	*Not supported*
H3a	Compared to problem-focused coaching, solution-focused coaching leads to higher approach goal orientation	*Supported*
H3b	Compared to problem-focused coaching, solution-focused coaching leads to lower avoidance goal orientation	*Not supported*
H4a	Compared to problem-focused questioning, solution-focused questioning will lead to stronger increases in goal attainment	*Not supported*
H4b	Compared to problem-focused questioning, solution-focused questioning will lead to more and higher quality action planning (i.e., number and quality of action steps) directly after the experimental coaching intervention	*Not supported*
H5a	Compared to problem-focused questioning, solution-focused questioning leads to higher *reported* problem-solving actions (i.e., extent of performing action steps) during follow-up	*Not supported*
H5b	Compared to problem-focused questioning, solution-focused questioning leads to higher goal attainment during follow-up	*Not supported*
H5c	Compared to problem-focused questioning, solution-focused questioning leads to higher *actual* problem-solving actions during follow-up	*Not supported*

See the main text for a description of the statistical results.

### Manipulation check

Results showed that the experimental manipulation was successful. First, participants in the problem-focused condition scored higher on the degree to which the experiment had instructed them to imagine their problem being strongly present than participants in the solution-focused conditions (*F*(2, 180) = 58.12, *p* < 0.001, see Table 1). Second, participants in the miracle condition scored higher on the degree to which the experiment had instructed them to image a situation in which their problem had suddenly disappeared than participants in other two conditions (*F*(2, 180) = 479.45, *p* < 0.001). Finally, participants in the success condition scored higher on the degree to which the experiment had instructed them to image what they had done before to solve the problem than participants in the other two conditions (*F*(2, 180) = 63.99, *p* < 0.001). Post hoc testing confirmed that differences between conditions were significant (all *p*’s < 0.001).

### Hypothesis testing

H1a predicted that the two solution-focused conditions (miracle and success) would elicit higher positive affect (i.e., hedonic tone, energetic arousal) than the problem-focused condition. Results yielded a significant main effect of condition for hedonic tone, *F*(2, 180) = 11.85, *p* < 0.001, η*p*2 = 0.12: participants in the solution-focused conditions reported significantly higher hedonic tone than participants in the problem-focused condition, *t*(180) = 4.81, *p* < 0.001. Similarly, results showed a significant main effect of condition for energetic arousal, *F*(2, 180) = 6.81, *p* = 0.001, η*p2* = 0.07: participants in the solution-focused conditions reported significantly higher energetic arousal than participants in the problem-focused condition, *t*(180) = 3.69, *p* < 0.001. Thus, H1a was supported.

H1b predicted that the two solution-focused conditions would elicit lower negative affect (i.e., tense arousal) than the problem-focused condition. Results showed a significant main effect of condition for tense arousal, *F*(2, 180) = 3.78, *p* = 0.025, η*p2* = 0.04: participants in the solution-focused conditions reported significantly lower tense arousal than participants in the problem-focused condition, *t*(180) = −2.75, *p* = 0.007. Thus, H1b was supported.

H2a predicted that the two solution-focused conditions would elicit higher self-efficacy than the problem-focused condition and H2b predicted that the success condition would elicit higher self-efficacy than the miracle condition. These hypotheses were not supported, *F*(2, 180) = 1.00, *p* = 0.368, η*p2* = 0.01.

H3a predicted that the two solution-focused conditions would elicit higher approach goal orientation than the problem-focused condition. Results showed a significant main effect of condition for approach goal orientation, *F*(2, 180) = 3.83, *p* = 0.024, η*p2* = 0.04: participants in the solution-focused conditions reported significantly higher approach goal orientation than participants in the problem-focused condition, *t*(180) = 2.65, *p* = 0.009. Thus, H3a was supported. H3b predicted that the two solution-focused conditions would elicit lower avoidance goal orientation than the problem-focused condition, but was not supported, *F*(2, 180) = 0.71, *p* = 0.494, η*p2* = 0.01.

H4a predicted that the two solution-focused conditions would yield a stronger increase in participants’ goal attainment after the coaching exercise than the problem-focused condition. Repeated measures analyses with time as within-subject variable and condition as between-subject variable revealed a significant main effect of time, *F*(1, 180) = 95.63, *p* < 0.001, η*p2* = 0.35. In all three conditions, participants reported higher goal attainment after the self-coaching exercise than before, all *p*’s < 0.001. The time x condition interaction was not significant, *F*(2, 180) = 1.45, *p* = 0.237, η*p2* = 0.02, indicating that participants’ increase in goal attainment did not differ between conditions. Results furthermore showed that there were no differences between conditions in participants’ goal attainment at the start of the manipulation nor in the severity of the problem they had described, both *p*’s > 0.05. Thus, H4a was not supported.

H4b predicted that the two solution-focused conditions would lead to more and higher-quality action planning than the problem-focused condition. The average number of action steps was the same in all conditions, *F*(2, 180) = 0.08, *p* = 0.926, η*p2* = 0.01^2^ and there was no difference between conditions for any of the four quality indicators (*specificity*: *F*(2, 176) = 2.63, *p* = 0.075, η*p2* = 0.03; *uniqueness*: *F*(2, 176) = 0.69, *p* = 0.505, η*p2* = 0.01; *behavior*: *F*(2, 176) = 0.59, *p* = 0.557, η*p2* = 0.01; *approach goal orientation*: *F*(2, 176) = 1.18, *p* = 0.309, η*p2* = 0.01). Thus, H4b was not supported.

H5a predicted that the two solution-focused conditions would lead to higher *reported* problem-solving actions within the period of 14 days after the experimental coaching intervention than the problem-focused condition. Results showed no differences between conditions in formulated action steps, *F*(2, 154) = 0.01, *p* = 0.992, η*p2* = 0.00, nor in the amount of effort spent on performing those action steps, *F*(2, 128) = 1.53, *p* = 0.221, η*p2* = 0.02. Additionally, a Pearson Chi-Square test showed that the proportion of participants who reported additional action steps (*n* = 22; 13.8%) did not differ as a function of condition *X*^2^ (2, *N* = 160) = 3.96, *p* = 0.138. Thus, H5a was not supported. H5b predicted that participants in the two solution-focused conditions would report higher goal attainment than participants in the problem-focused condition. We found no support for this hypothesis, *F*(2, 157) = 0.375, *p* = 0.688, η*p2* = 0.01.

H5c predicted that participants in the two solution-focused conditions would show higher *actual* problem-solving actions (i.e., website visits). We found no support for this hypothesis: a Pearson Chi-Square test indicated that participants from all three conditions^3^ visited the website equally, *X*^2^ (2, *N* = 154) = 1.39, *p* = 0.499.

## Discussion

Despite the popularity of coaching for increasing well-being and thriving at both the workplace and the private domain, research has lacked behind in uncovering the mechanisms behind coaching effectiveness. Specifically, only little is known about the effectiveness of specific type of coaching questions, and it has remained unclear if the positive effects of such questions can be sustained outside of coaching sessions. The current study therefore examined the immediate effects of solution-focused and problem-focused coaching techniques in an experimental setting and investigated if these questions led to goal-directed changes during a brief follow-up period of 14 days. We showed that when implemented in a self-coaching writing exercise, solution-focused questioning –a popular approach to the practice of coaching– fosters affective self-regulation relatively more than problem-focused questioning. That is, solution-focused questioning promotes positive emotions, hampers negative emotions, and increases people’s motivation to solve their problem (i.e., approach goal motivation). Yet, solution-focused questioning was not more effective than problem-focused questioning in reducing avoidance goal orientation or in promoting self-efficacy, action planning, problem solving and goal attainment. In fact, both solution- and problem-focused questioning increased perceptions of goal attainment right after the writing exercise and after a period of 14 days. Below, we will discuss our findings and their implications in more detail.

Our results show that thinking about solutions rather than problems makes people not only feel good, but also motivates them to strive for gains while keeping an eye on potential losses. That is, solution-focused questioning stimulated approach motivation but did not simultaneously inhibit avoidance motivation. A possible explanation for this finding might be that approach and avoidance motivation are relatively independent concepts (Elliot and Covington, 2001), and are therefore influenced through different systems. It might also be possible that investing in solutions for complex problems –that often are systemic and not entirely within one’s control– is only adaptive when the problem will not get worse. In that case, adopting a prevention strategy (i.e., concerned with assuring safety and avoiding negative outcomes) can provide some degree of control (Higgins, 1997).

Contrary to our expectations and earlier empirical findings, we did not find that solution-focused questioning was more effective than problem-focused questioning in increasing people’s self-efficacy beliefs, nor did we find any differences between the miracle and the success question in that regard. This is surprising, given that previous success experiences are deemed the most important source of self-efficacy (Bandura, 1977). We see two explanations for this unexpected finding. First, it is possible that the success experiences made salient during the coaching exercise were too broad to be a credible source for solving one’s current problem. While mastery experiences in one domain can lead to spill-over effects to other domains, meaning that previous successes and associated positive experiences for example at work may boost motivation and positive affect to approach problems in private life, this is only the case if the same skills are required (e.g., general self-management strategies, Bandura, 2006). The skills that participants recalled during the coaching exercise may thus not have fully matched the skills needed to solve their current problem. It is particularly important for solution-focused coaches to not blindly focus on clients’ strengths but to enable clients to transfer the right prior experienced skills to the current problem. Second, the problems that participants expressed were complex and at least partly contextual (see the Supplementary material), which may mean that participants may have had situational restrictions in mind when reflecting on their ability to solve the problem. Indeed, Table 1 shows moderate self-efficacy beliefs and relatively low variance among participants in all three conditions.

Another unexpected finding was that problem-focused questioning was equally successful as solution-focused questioning in promoting goal attainment (i.e., how close people felt to solving their problem). Interestingly, this was still evident in all groups 14 days later. Although ruminating on problems can be damaging to clients’ immediate affective states, taking time to reflect on one’s problem may still feel like progress. According to the Transtheoretical Model of Change (Prochaska et al., 2015), people need to become aware of their problem, its causes and consequences, before they are ready to act. Although the awareness of a problem can be uncomfortable (reduced positive affect – a finding we also see in our study), it is a crucial first step on the road to change and may facilitate rather than impede problem-solving actions when one stops digging into the problem in time.

Lastly, the results showed no differences between problem-focused and solution-focused questioning with regard to people’s immediate action planning (i.e., number and quality of action steps), and their *reported* and *actual* problem-solving actions. In other words, thinking about solutions rather than problems did not make people actually *do* more to solve their problem. Our self-coaching writing exercise, in which participants were asked to describe a problem and reflect on it, may have been a push to start acting on the problem, irrespective of the experimental condition they were in. Thus, raising the salience of a problem may already trigger action planning and subsequent actions. Alternatively, the effects of problem-focused and solution-focused questioning techniques may outweigh each other in promoting or hampering action taking. While problem-focused questioning may cause deep reflection but also deactivating negative moods such as sadness and weariness (see Kreemers et al., 2020), solution-focused questioning may cause divergent thinking but also unrealistic fantasies that hinder the planning of concrete actions. Unlike concrete goals, positive fantasies lack a clear commitment to behavior (Oettingen, 2012). When indulging in positive thoughts, one can easily forget that this positive future has not been realized yet, which ultimately hinders goal striving and pursuit.

### Theoretical implications

The results of the present study provide a better understanding of the effects of questioning techniques in coaching and advance the literature in several ways. First, we answered to the call for a broader understanding of the psychological mechanisms that render positive coaching outcomes (Bachkirova and Kauffman, 2009). We shed light on the most essential tool that coaches have: asking questions. We showed that solution-focused questions are more effective than problem-focused questions when the goal of coaching is to make people feel good, and to help people strive toward solving their problem (rather than preventing it from getting worse). For factors deemed essential for goal-directed self-regulation, the type of questioning made no difference.

Second, by examining the effects of questioning techniques on participants’ problem-solving actions during a brief follow-up period, we uncovered their differential potential to alter behavior – the ultimate goal for many clients and their coaches. Specifically, we showed that solution-focused and problem-focused questioning did not lead to different behavioral outcomes during this period. Thus, although a strength-based approach in coaching seems particularly useful in stages in the coaching process where clients get lost in complex rumination and feelings of despair, this approach may be insufficient for sustaining behavioral change. More theory development and research are needed to better understand which interventions have which effects in the different temporal stages of coaching (see also Theeboom et al., 2017).

Finally, while prior research with university students showed that individuals benefited more from solution-focused than problem-focused questioning, this finding was only partly replicated in our study with medical residents. This can be explained by the differences in samples: the type of problems that medical residents face in their job may fundamentally differ from those of students (e.g., study-related stress, Theeboom et al., 2016) in magnitude and complexity. First, the problems of employees and students may differ in *magnitude*. Theeboom et al. (2016) speculated that students’ problems might not be pressing enough. For example, students were instructed to think about problems that were “frustrating for them” or were posing a “dilemma […] where [they] feel caught between two or more possible courses of action” (e.g., Grant and Gerrard, 2020). These types of problems were probably less severe than the problems mentioned by the healthcare workers in our sample. Second, the problems of employees and students may differ in *complexity.* Healthcare workers are part of large organizational systems in which they can have limited autonomy and control in their work. The work-related problems they face may often involve structural organizational factors (hindrance stressors) and significant others (e.g., colleagues, supervisors or patients), which can significantly impact their perception of behavioral control, motivation and options for problem-solving actions (Yang and Li, 2021). At the same time, the job demands (e.g., high workload, emotional demands) faced by the residents in this study might at least partly overlap with the experience of employees from relevant other settings (e.g., education). Consequently, we expect the findings to be generalizable across other professions outside of healthcare. All in all, it is possible that both the severity and complexity of the problems that coaching clients aim to solve influence the effectiveness of coaching questions for outcomes such as self-efficacy, goal attainment, and action planning and behavior. Therefore, as experimental studies encompass only a one-time and short (although controlled) intervention, future research could further improve its ecological validity by examining the effects of coaching questioning techniques in real coaching sessions. After all, coaching is a process.

### Practical implications

Asking (the right) questions is an essential part of coaching. Our results show that not all types of questions are equally effective. Coaching questions that convey a positive outcome make the client feel good and motivate them to pursue their goals whereas “problem talk” goes along with unpleasant feelings. In coaching practice, it would be neither desirable nor constructive to eliminate the problem from the coaching conversation altogether. However, if coaches –in a specific stage of the coaching process– aim to reinforce positive feelings and inspire optimism and hope for the future, they might do well to ask solution-focused questions. This may help clients to temporarily detach from their problem and develop a different and broader view on their situation.

Second, our results suggest that feeling good is a “nice-to-have” rather than a “must-have” for clients to pursue and achieve their goals: with positive outcomes in mind, people feel better in the short run, but these immediate affective reactions may not translate into goal-directed behaviors in the long run. Thus, asking solution-focused questions is not necessarily helpful in every stage of the coaching process. Given that coaching clients enter a coaching session with a description of what brought them to seek support in the first place (the preparatory contemplation stage of the coaching process), focusing on the problem at hand often is the logical first step. Especially when clients want to talk about their problems –which can be a cathartic experience– coaches should meet this need and not counter it with a rigid focus on solutions (Theeboom et al., 2016). Coaching is typically a blend of solution-focused and problem-focused techniques (Grant, 2012), and not one or the other.

Finally, we recognize that effective questioning is only one pillar of successful coaching conversations. While skillfully asked questions can fundamentally set the tone of a coaching conversation by provoking thinking and self-awareness, the ultimate goal of coaching is client development and change. Therefore, coaches need to assist their clients in setting concrete and attainable goals and turning intentions into actions – one of the biggest challenges for many clients.

### Limitations and future research

Our study is not without limitations. First, the experimental design of our study did not allow us to capture the coaching process in all its complexity. However, it afforded experimental control by which we could compare the pure effects of different questions techniques unaffected by relational (and other) factors that influence coaching outcomes in real-life. It is important to note that participants engaged in a short, online self-coaching exercise rather than a real coaching session with a professional coach. Real-life coaching is a joint and complex behavioral change process together with a professional that is different in many ways from self-coaching where such a professional is absent. While our design allowed us to disentangle the effects of coaching questions from other factors that play a role during coaching, a necessary next step is to investigate and extend the current findings using more ecologically valid procedures. Having said this, we are confident that our participants took the online exercise seriously as became clear from their serious and extensive responses to the open questions. Additionally, given that coaches regularly use (written) homework exercises for their clients between sessions, our results stress the (potential) benefits of such practice.

Second, we realize that the distinction between solution- and problem-focused questioning is in part artificial, and that real-life coaching is a mixture of many different approaches –of which solution- and problem-focused coaching are merely two– rather than the strict following of one single approach. Yet, disentangling the effects of both coaching approaches, can inform coaching practitioners of the unique effects that different types of questions may have on their clients.

We suggest some promising directions for future research. Based on the finding that a short self-coaching writing exercise could already increase perceptions of goal-directed change over time, it would be interesting to explore to what extent these perceptions are related to concrete behaviors (e.g., action planning and execution). Given that coaching tends to be an expensive enterprise, shortcuts to goal attainment could allow clients with fewer financial resources to benefit from coaching as well. Finally, as to gain an in-depth understanding of what happens in and leads to successful coaching, extensive process studies are needed that combine coach- and client perspectives and ultimately relate them to coaching outcomes. Such insights into the process of coaching will not only advance the theory of coaching but will also inform coaching practice in important ways. If coaching as a profession is seeking to move beyond an “anyone can coach” – approach, it is important to know which (trained) coaching skills –including question techniques– are essential in which stage of the coaching process for attaining coaching goals.

## Conclusion

In this study we compared the effectiveness of solution-focused and problem-focused questions in driving positive outcomes of coaching. Our study shows that thinking about solutions rather than problems during a self-coaching writing exercise increases both people’s immediate affective states and their goal-directed motivation. Both approaches, however, are equally effective for immediate action planning and execution during a brief follow-up period. Further research is needed that examines the variety and effectiveness of coaching questions in different stages of the coaching process.

## Data availability statement

Given restrictions from the ethics review board and considering that sensitive personal data are handled, it is not possible to make the data freely available. The data that support the findings of this study are available from the corresponding author, LS, l.solms@uva.nl, upon request.

## Ethics statement

The study involving human participants was reviewed and approved by the Ethics Review Board of the University of Amsterdam (2020-WOP-12154). The participants provided their written informed consent to participate in this study.

## Author contributions

LS, AV, JK, TT, and BB coded the qualitative data. LS, AV, and JK analyzed the data. LS drafted the manuscript. All authors were involved in the conception and design of the study as well as the collection and interpretation of the data, reviewed and approved the manuscript.

## Footnotes

1 We are aware that the term problem-focused coaching is used primarily by advocates of solution-focused coaching. Real-life coaching often uses a blend of solution- and problem-focused techniques (Grant, 2012) but since we are interested in the effects of specific coaching questions, we separate them in our study design.

2 Log-transformation of the data showed comparable results.

3 For 9 participants, it was not possible to retrieve if they had visited the website within the set period of 10 days. Because exclusion of these participants did not lead to changes in the results, we included their data in the final analyses.
