# Supplementary material for: Simply effective? The differential effects of solution-focused and problem-focused coaching questions in a self-coaching writing exercise
Source: Front Psychol. 2022 Aug 18;13:895439. doi: 10.3389/fpsyg.2022.895439 (PMC9435469; doi:10.3389/fpsyg.2022.895439)
Supplement: Supplementary file 1 [file Data_Sheet_1.docx]

Supplementary Material

# Self-coaching writing exercise

Participants in all three conditions took part in an online self-coaching writing exercise. First, in all three conditions, participants answered the questions in the general instruction (see supplementary Table 1). Then, participants answered either solution-focused or problem-focused coaching questions, depending on the condition to which they had been assigned. Supplementary Table 2 displays the instructions that followed the general instructions in the experimental conditions.

| **Supplementary Table 1** | |
| --- | --- |
| ***General instruction*** | |
| 1. | *This is an important part of the study. Please take a moment to think about this.*  *First, we would like to ask you to describe the problem in about 50-100 words answering the questions below.*  *You can formulate your answers in the indicated text fields below each question.*  What is the problem? |
| 2. | On a scale from 1-10, to what extent is the problem causing discomfort (1 = *no discomfort at all*, 10 = *heavy discomfort*)? |
| 3. | In what ways are you affected by the problem? Think for example of feelings, or thoughts that arise in connection with the problem, but also how the problem might affect your functioning at work or in your private life. |
| 4. | On a scale from 1 to 10, to what extent have you currently reached the solution to this problem? You can give your answer by moving the slider below (1 = *solution not reached at all*, 10 = *solution reached*) |
| *Note.* To answer question 3, participants were asked to use approximately 50 words. For all open questions (i.e., question 1 and 3) participants were asked to formulate their answers in text fields presented below each question. | |

| **Supplementary Table 2** | |
| --- | --- |
| ***Problem-focused questions*** | |
| 1. | *Now comes the most important part of the study. Take a moment to think about the following:*  *Think of a situation in the past where your problem was strongly present.* |
| 2. | Thinking back to that day when your problem was strongly present, what is the first thing that you notice? |
| 3. | How did you behave in this situation in the past where the problem was strongly present? |
| 4. | What thoughts did you have in this situation in the past where the problem was strongly present? |
| 5. | How did you feel in this situation in the past where the problem was strongly present? |
| 6. | How did others notice that the problem was strongly present in this situation in the past? |
| ***Solution-focused miracle questions*** | |
| 1. | *Now comes the most important part of the study. Take a moment to think about the following:*  *Imagine that tonight you go home and go to sleep. At night, however, a miracle happens. You have no idea how, but the next day when you get up you notice that that day your problem is no longer or barely present, and your desired situation has already come about. As the day progresses, the latter becomes more and more evident.* |
| 2. | What is the first thing you notice when you wake up? |
| 3. | How do you behave in this situation where the problem has suddenly disappeared? |
| 4. | What thoughts do you have in this desired situation where the problem is suddenly no longer there? |
| 5. | How do you feel in this desired situation where the problem has just disappeared? |
| 6. | How do others notice that the desired situation has come about because the problem is no longer present? |
| ***Solution-focused success questions*** | |
| 1. | *Now comes the most important part of the study. Take a moment to think about the following:*  *Think of a situation in the past where you ran into the problem, but where you managed to (nearly) solve the problem.* |
| 2. | Thinking back to that day when you managed to (nearly) solve your problem, what is the first thing that you notice? |
| 3. | How did you behave in this situation in the past when you managed to (nearly) solve the problem yourself? |
| 4. | What thoughts did you have in this situation in the past where you managed to (nearly) solve the problem yourself? |
| 5. | How did you feel in this situation in the past where you managed to (nearly) solve the problem yourself? |
| 6. | In this situation in the past, how did others notice that you had (nearly) solved the problem? |
| *Note.* To answer questions 2 to 6, participants were asked to use approximately 50 words. For all open questions (i.e., question 2 to 6) participants were asked to formulate their answers in text fields presented below each question. | |

# Action planning at T1: description of the quality criteria and the coding process

We recorded the number and quality of action steps of each participant. Some participants described action steps that were largely the same, such as “deciding what tasks are my priority and what tasks aren’t” and “choosing to postpone certain tasks”. We therefore first removed a participant’s redundant action steps and then counted the number of action steps per participant. The quality of action steps was assessed by means of four indicators: *specificity*, *uniqueness*, *behavior*, and *approach goal orientation*.

*Specificity* concerns the degree to which action steps were concrete (as opposed to vague / undefined). Examples of concrete action steps are “make decisions independently and then evaluate with my supervisor whether I have taken these decisions correctly” or “discuss during my performance review that the current internship is actually too heavy”. Examples of vague / undefined action steps are ”reflecting” or “better listening”. All action steps were coded on a four-point scale ranging from 0 (*not concrete at all*) to 4 (*very concrete*).

*Uniqueness* refers to the extent to which action steps belonged to a unique category of actions within the set of action steps a participant had described. Examples of unique action categories are ‘time management’ or ‘communication with a relevant other’. An unique action step was coded as 1 whereas an action step that belonged to the same category as a participant’s other actions was coded as 0. For example, “plan time for myself to relax/exercise” and “create a day schedule with small goals per day” were coded as unique actions steps, whereas ”do not continue working at home” and “work as little overtime as possible” were coded as belonging to the same category (i.e., regulation of working hours).

*Behavior* concerns the extent to which action steps reflected behavior rather than cognitions (thoughts). Examples of behavioral action steps are “going for a run” or “making an appointment with my supervisor”. Examples of action steps that reflect cognitions are “thinking about my future job” or “analyzing my problem”. Behavioral action steps were coded as 1, all other action steps were coded as 0.

*Approach goal orientation* concerns the extent to which action steps reflected an approach (as opposed to avoidance) orientation. An example of an approach-oriented action step is “discuss this problem with colleagues for tips”. An example of an avoidance-oriented action step is “trying to look at myself less judgmentally”. Approach-oriented action steps were coded as 1, whereas avoidance-oriented action steps were coded as 0.

In pairs of two, the authors conducted the coding of the quality indicators based on the above-described coding scheme, after first having discussed the coding scheme with each other. After coding a subset of the responses (i.e., responses of the first 60 participants), we calculated the interrater reliability (i.e., Cohen’s kappa) between the two coders for each quality indicator. If the interrater reliability was not yet sufficient (below .70), the two coders discussed their scoring and, if necessary, revised the coding scheme. Hereafter, they continued coding a second subset (or the complete set) of responses. The two coders discussed remaining differences and came to a unanimous coding decision.

# Additional measures: the effects of solution-focused and problem-focused coaching questions on cognitive flexibility

Next to the outcomes discussed in the main paper, we included an additional outcome, that is cognitive flexibility. Cognitive flexibility refers to an individual’s capacity to flexibly process information and making use of flat associative hierarchies (De Dreu et al., 2011) in pursuit of a solution or goal. This skill is particularly valuable for coaching clients that need to break loose from traditional ways of thinking and explore alternative ways to approach their problem. Coaches can use specific techniques, such as the miracle question, to help their clients to think out of the box. By stimulating the client to think about a world where the problem is absent, people can experience themselves in uncharted waters freed of the restrictions tied to the problem. Theeboom and colleagues (2016) found that solution-focused questioning (as opposed to problem-focused questioning) led to more cognitive flexibility in a sample of students that completed a self-coaching exercise. In this study we examined whether this finding was replicated in a sample consisting of working adults. Additionally, we aimed to investigate the effects of an alternative solution-focused coaching question, that is the success question. In the success question, people are instructed to think back to previous behaviors that have helped in managing the problem. Although we expect that the success question – just as the miracle question – stimulates associative thinking, the focus is restricted to past behaviors. Consequently, we expected that people in the miracle condition will experience more cognitive flexibility because of their unrestricted attentional focus.

In this study, cognitive flexibility was measured with the Category Inclusion Task (CIT; Rosch, 1975), as in prior coaching research (Theeboom et al., 2016). Participants were asked to rate how prototypical objects are for a particular category using a 10-point scale ranging from 1 (*doesn’t belong into this category and does not resemble at all*) to 10 (*belongs into this category and resembles a lot*). Participants rated three examples in four categories that were either strong, intermediate, or weak examples of the category. We used vehicles, furniture, vegetables, and clothing as the categories, with bus, airplane, camel (vehicles), couch, lamp, telephone (furniture), carrot, potato, garlic (vegetables), and skirt, shoes, handbag (clothes) as the strong, intermediate, and weak examples of the category.

*Hypothesis A1*: Compared to problem-focused questioning, solution-focused questioning leads to higher cognitive flexibility (a). Compared to the solution-focused success question, the solution-focused miracle question leads to higher cognitive flexibility (b).

## Results

We predicted that the solution-focused miracle condition and the solution-focused success condition would elicit higher cognitive flexibility than the problem-focused problem condition (HA1a) and that the solution-focused miracle condition would elicit higher cognitive flexibility than the solution-focused success condition (HA1b). We found no support for these hypotheses. *F*(2, 180) = 0.40, *p* = .672, *ηp2* = 0.04. Participants in both the miracle (*M* = 4.70, *SD* = 1.64) and the success condition (*M* = 4.50, *SD* = 1.25) did not report higher cognitive flexibility than participants in the problem-focused condition (*M* = 4.54, *SD* = 1.10).

# Problem description: examples

*Example 1*: ‘The workload is high. We have long days of surgery and are working in a small group, so the pressure is high. There is quite a generation gap between the senior specialists, and the younger generation of residents. Many of us, including me, wonder how sustainable the profession is in its current form, as 'nowadays' things like family life become more important than they used to be. It is difficult to talk about this with the older generation.’

*Example 2*: ‘My supervisors are hardly involved in my training and I receive little to no guidance.’

*Example 3:* ‘As an inexperienced assistant, there are many things I don't know. Nurses expect me to know everything. How to deal with this? Saying you don't know something doesn't seem to be received positively. But pretending to know when you don't doesn't feel right.’

**5** **References**

De Dreu, C. K. D., Nijstad, B. A., & Baas, M. (2011). Behavioral activation links to creativity because of increased cognitive flexibility. *Soc. Psychol. Personal. Sci.* 2, 72-80. doi: 10.1177/1948550610381789

Theeboom, T., Beersma, B., & Van Vianen, A. E. (2016). The differential effects of solution-focused and problem-focused coaching questions on the affect, attentional control and cognitive flexibility of undergraduate students experiencing study-related stress. *J. Posit. Psychol*. 11, 460-469. doi: 10.1080/17439760.2015.1117126

Rosch, E. (1975). Cognitive representations of semantic categories. *J. Exp. Psychol. Gen.* 104, 192-233. doi: 10.1037/0096-3445.104.3.192
